# Health psychology and the coronavirus (COVID‐19) global pandemic: A call for research

**DOI:** 10.1111/bjhp.12414

**Published:** 2020-03-30

**Authors:** Madelynne A. Arden, Joseph Chilcot

**Affiliations:** ^1^ Centre for Behavioural Science and Applied Psychology Sheffield Hallam University UK; ^2^ Institute of Psychiatry, Psychology and Neuroscience King's College London UK

On 11 March 2020, the World Health Organization (WHO) declared COVID‐19, a disease caused by coronavirus SARS‐CoV‐2, a pandemic. At the time of writing, there are significant outbreaks across much of the world with Europe identified as the epicentre. It seems clear that health psychology has an important role to play in understanding how people will respond and cope to the threat of COVID‐19 and how they will adhere and adapt to transmission‐related behaviours including hand washing and self‐isolation.

Efforts to control and reduce coronavirus transmission rely on behavioural change and maintenance. In 2010, *BJHP* published a review of ‘Demographic and attitudinal determinants of protective behaviours during a pandemic’ (Bish & Michie, 2010), finding that communications to promote preventative behaviours should be targeted to specific demographic groups and should focus on raising perceived threat and the effectiveness of behavioural measures to reduce risk. Susan Michie and colleagues have recently written about understanding behaviour and behavioural strategies for reducing COVID‐19 transmission (https://blogs.bmj.com/bmj/2020/03/03/behavioural‐strategies‐for‐reducing‐covid‐19‐transmission‐in‐the‐general‐population/ and https://blogs.bmj.com/bmj/2020/03/11/slowing‐down‐the‐covid‐19‐outbreak‐changing‐behaviour‐by‐understanding‐it/) drawing on the COM‐B model (Michie *et al.*, 2011) to identify issues of capability, opportunity, and motivation that might impact on behaviours and discussing behavioural strategies to address barriers.

Beyond behaviour change, health psychology also has a role in understanding how people might respond to and cope with the threat of a global pandemic and changes to their lives that are made in an effort to reduce that threat. The Economic and Social Research Institute in Dublin has recently produced a working paper on 'Using behavioural science to help fight the coronavirus' (Lunn *et al.*, 2020 https://www.esri.ie/system/files/publications/WP656.pdf) which in addition to personal hygiene behaviours, considers pro‐social behaviours, panic‐buying, communication, risk perception, and the impacts of isolation. In 2013, *BJHP* published the findings of a survey conducted during the H1N1 influenza pandemic in 2009 that indicated the importance of precise and clear information about control measures for reducing anxiety (Taha *et al.*, 2014). Brooks *et al. *(2020) recently published a rapid review focusing on the psychological impact of quarantine and how best to enable people to cope which reported the importance of clear information, and that voluntary quarantine is ‘associated with less distress and fewer long‐term complications’.

There is also a need to understand the potential physical and psychosocial impact of COVID‐19 on front‐line health care staff. Brooks *et al. *(2018) undertook a systematic review of the factors affecting the psychological well‐being of health care workers involved in the severe acute respiratory syndrome (SARS) crisis and recommended that employers should provide support for those most at risk and that health care workers should be prepared for the potential psychological impact of this work. It seems likely that these impacts will be significant in the case of COVID‐19.

Despite similarities with previous pandemics and a rapid response by the scientific community to understand COVID‐19 and reduce its global impact, there is still much that we do not know, especially given the novel features of COVID‐19, and governments varying responses to the crisis worldwide. There is therefore an urgent need for health psychology research. To that end, we invite contributions to *BJHP* that address any area of health psychology research related to the coronavirus pandemic to our fast‐track COVID‐19 route. We will prioritize and expedite the peer review process for these papers so that they can be published online without delay. Papers may be of any of the traditional forms described in our author guidelines, but we will also accept brief reports on this topic. Brief reports should not exceed 2,000 words and should have no more than two tables or figures (see online author guidelines, ‘Brief‐Report COVID‐19’, for more details).
